# Financial Stress and Smoking within a Large Sample of Socially Disadvantaged Australians

**DOI:** 10.3390/ijerph14030231

**Published:** 2017-02-25

**Authors:** Ashleigh Guillaumier, Laura Twyman, Christine Paul, Mohammad Siahpush, Kerrin Palazzi, Billie Bonevski

**Affiliations:** 1 School of Medicine & Public Health, University of Newcastle and Hunter Medical Research Institute, CBMHR, P.O. Box 833, Newcastle, NSW 2300, Australia; laura.twyman@newcastle.edu.au (L.T.); chris.paul@newcastle.edu.au (C.P.); billie.bonevski@newcastle.edu.au (B.B.); 2 Department of Health Promotion, Social and Behavioral Health, College of Public Health, University of Nebraska Medical Center, Omaha, NE 68198, USA; msiahpush@unmc.edu; 3 Clinical Research Design IT and Statistical Support Unit, University of Newcastle & Hunter Medical Research Institute, Newcastle, NSW 2300, Australia; kerrin.palazzi@hmri.org.au

**Keywords:** smoking, disadvantage, financial stress

## Abstract

Financial stress is associated with fewer quit attempts and higher relapse rates. This study aimed to compare financial stress among smokers, ex-smokers and never smokers in a highly socioeconomically disadvantaged sample. The study also aimed to determine whether specific indicators of financial stress differ according to smoking status. Adult clients seeking welfare assistance from two Social and Community Service Organisation sites in New South Wales, Australia, were invited to complete a cross-sectional survey between March 2012 and December 2013. Responses to a financial stress scale, smoking status and demographics were collected. Linear and logistic regression modelling was used to examine associations between smoking status and financial stress. A total of 1463 participants completed the survey. Current smokers had significantly higher total financial stress scores than ex-smokers and non-smokers respectively. Current smokers also had higher odds of severe financial stress indicators, such as going without meals (Odds Ratio = 2.2 and 2.0), than both non-smokers and ex-smokers. Even among a highly socioeconomically disadvantaged sample with high levels of financial stress, smoking status further exacerbates experiences of deprivation. Given the relationship between financial stress, socioeconomic disadvantage and difficulty quitting, it is important to provide enhanced cessation support to smokers experiencing financial stress.

## 1. Introduction

Financial stress refers to the experience of economic hardship and may include financial anxiety, being in debt and being unable to afford consumer items such as food and clothing [1]. Among the general population, smokers experience higher financial stress than non-smokers, and ex-smokers with high financial stress are more likely to relapse [2]. Although smoking is a significant correlate of financial stress [1], it is more often experienced by those with a low socioeconomic status (SES) [3]. Additionally, within smoking households, higher percentages of the total household expenditure on tobacco and the experience of financial stress are positively related [1]. Smoking households also have higher odds of experiencing ‘severe’ financial stress than non-smoking households, marked by individual indicators such as being unable to heat the home, going without meals or seeking assistance from welfare or community organisations [1].

The experience of financial stress is also associated with smoking cessation. Findings from the International Tobacco Control Four Country Survey (Australia, Canada, UK, US) indicate that smokers with high financial stress are less likely to make a quit attempt and less likely to maintain abstinence when they do make a quit attempt [4]. Indeed, in a US study of racially/ethnically diverse smokers of predominantly low SES, those with greater levels of financial strain at baseline had significantly reduced odds of abstinence at 26 weeks post-quit [5]. However, when smokers do quit, their financial stress decreases [3].

Given that low SES populations are more likely to experience higher levels of financial stress, and this is negatively associated with cessation, there is a clear need to focus on vulnerable and highly disadvantaged groups within the financial stress literature; particularly as across western countries, smoking rates are highest among socially and economically vulnerable and disadvantaged groups. Low SES groups tend to face a number of social and economic hardships, which can be exacerbated by expenditure on tobacco. For example, countries such as Australia and New Zealand consistently implement policies increasing tobacco taxation. Increases in taxes that raise the price of tobacco are an effective measure for reducing smoking prevalence, especially in lower SES groups [6], however they also increase the financial stress experienced by these smokers who are least equipped to deal with it [7]. In other social and political contexts, such as the UK, welfare for the most disadvantaged is being cut, leaving the poorest with lower incomes in real terms. While lower SES smokers are more likely to use price-minimising strategies to continue to afford tobacco [8], in the face of rising tobacco prices and increased cost of living, they are also likely to engage in behavior that further exacerbates their circumstances of deprivation [9].

Exploring and understanding the relationship between social disadvantage and financial stress is important to aid the development of programs to address smoking, which may reduce financial hardship and improve material wellbeing in highly disadvantaged groups. The aim of this study was to compare overall financial stress among smokers, ex-smokers and never smokers in a very socioeconomically disadvantaged sample. Furthermore, the study aimed to assess individual indicators of financial stress to determine whether specific experiences of financial stress differ according to smoking status among a very low SES sample.

## 2. Materials and Methods

### 2.1. Study Design

Secondary data analysis was undertaken using two data sets from two cross-sectional touchscreen computer surveys, both examining smoking in disadvantaged populations. One survey was conducted between March and December 2012 [10,11], the other between February 2012 and December 2013. Both surveys received ethical approval from the University of Newcastle’s Human Research Ethics Committee (approval numbers H-2011-0276 and H-2010-1002).

### 2.2. Setting and Sample

Participants were clients of a social and community service organisation (SCSO) recruited via two site locations under the same management in a disadvantaged area of Sydney, in New South Wales (NSW) Australia. Eligible participants were attending appointments for the receipt of welfare and financial aid (i.e., food vouchers, grocery items, bill payments), aged 18 years or over, able to comprehend English and healthy enough to give informed consent (as judged by organisation staff).

### 2.3. Recruitment and Data Collection

Recruitment and data collection were identical in both studies. Interested clients completed a survey assisted by research assistants who provided a written information statement and gained informed consent. Participants received an AUD$20 grocery card.

### 2.4. Measures

#### 2.4.1. Financial Stress

The financial stress scale [3] assesses participants’ experience of financial stress in the past six months. Scores on this scale range from zero to eight, with higher values indicating higher levels of financial stress. Participants were presented with the question stem “In the past six months, did any of the following happen to you because of a lack of money?” and asked to indicate “yes” (1) or “no” (0) to a list of items: (a) could not pay electricity, gas, or telephone bills on time; (b) could not pay the mortgage or rent on time; (c) pawned or sold something; (d) went without meals; (e) unable to heat or cool the home; (f) asked for financial help from friends or family; (g) asked for help from a welfare/community organisation. A response of “yes” indicated an experience of financial stress. Participants were also presented with the yes/no question, “Could you raise AUD$2000 in a week if there was an emergency?” where “no” was coded as the experience of financial stress.

#### 2.4.2. Smoking Status

Smoking status was assessed using two items: “Do you currently smoke tobacco products?” (yes, daily/yes, at least once a week/yes, but less often than once a week/no, not at all) and “Have you smoked at least 100 cigarettes or a similar amount of tobacco in your life?” (yes/no/not sure). Participants who smoked daily, or, who smoked occasionally (weekly or less) along with having smoked 100 cigarettes in their life were defined as current smokers. Ex-smokers were defined as not smoking currently but having smoked 100 cigarettes or a similar amount in their lives. Never smokers were defined as those reporting not currently smoking tobacco products and not having smoked 100 cigarettes or a similar amount in their lives.

#### 2.4.3. Demographic Characteristics

Age, gender, highest level of education, Indigenous status, housing status, marital status, personal weekly income, income source, number of adults in the household and number of children in household were assessed.

### 2.5. Statistical Analysis

Statistics of socio-demographic and smoking characteristics are presented by counts and percentages for categorical variables and by mean (standard deviation) or median (interquartile range) for continuous variables, depending on distribution. Comparison of characteristics between non-, ex-, and current smokers were performed using Chi-squared (categorical), ANOVA or Kruskal–Wallis (continuous) tests as appropriate.

Linear regression was used to examine the association between smoking status and financial stress (total score). Binary logistic regression was used to examine the association between smoking status and each of the financial stress questions (Yes/No). Potential confounders included in modelling were selected a priori, based on clinical knowledge and included age, gender, education, indigenous status, number of adults in house, number of children in house and income level.

Collinearity of variables was checked using variance inflation factors and linearity assumption for continuous variables and the outcomes were examined. Crude and adjusted Least Square-Means (95% Confidence Interval, CI) and *p*-values are presented. Significance was set a priori at *p* < 0.05; SAS 9.4 (SAS Institute Inc., Cary, NC, USA) was used for all analyses.

## 3. Results

### 3.1. Sample

The demographic characteristics of the sample (*N* = 1463) are presented in Table 1. A total of 979 participants identified as current smokers (67%). The majority of the sample had not completed secondary schooling (73%), received a government pension or benefit as their primary source of income (91%), and half resided in supported or government housing (48%). The majority of the sample reported very low income of <$400/week (74%), which was on or below the poverty line of $390/week for a single person living in Australia during the time of survey [12]. Additionally, 16% of participants identified as indigenous, compared to the 2.9% of the population in NSW [13].

### 3.2. Financial Stress and Smoking Status

The association between smoking status and total financial stress score was examined and presented in Table 2. Adjusted analyses showed that current smokers had a higher total financial stress score than both non-smokers and ex-smokers (*p* < 0.001 and *p* = 0.002, respectively); non-smokers did not have a significantly higher total financial stress score than ex-smokers (*p* = 0.198).

When examining the individual financial stress items, adjusted analyses showed that current smokers had higher odds of pawning/selling something (Odds Ratio, OR = 2.8, 95% CI = 2.1–3.7, and OR = 1.9, 95% CI = 1.4–2.7 respectively), going without meals (OR = 2.2, 95% CI = 1.7–2.9 and OR = 2.0, 95% CI = 1.4–2.8 respectively) and asking for help from welfare organisations (OR =2.3, 95% CI = 1.5–3.5 and OR = 1.7, 95% CI = 1.0–2.9) than both non-smokers and ex-smokers (see Table 3). Current smokers also had higher odds of asking for help from family/friends than non-smokers (OR = 1.8, 95% CI = 1.3–2.4).

## 4. Discussion

The study found that although there was a high level of financial stress overall in this sample of socioeconomically disadvantaged individuals, current smokers exhibit higher levels of total financial stress compared with ex- and non-smokers. Further to this, current smokers had higher odds of reporting a range of specific indicators of financial stress in the past 6 months due to a shortage of money. In particular, smokers had higher odds of reporting individual indicators that are markers of severe financial stress, including going without meals and asking for help from welfare/community organisations.

The results of this study, demonstrating that smokers had higher levels of financial stress than ex- and non-smokers, contribute to a pattern consistent with past research [2,3,4]. Importantly, the current study adds to the literature by showing that smoking status is significantly associated with increased experiences of severe financial, social and material hardship faced by highly disadvantaged people. This supports the call by advocacy groups for smoking to be considered as a social justice issue, and as such addressed within the welfare sector. Currently in Australia, smoking and tobacco use are not addressed as part of routine care among welfare, mental health or substance abuse treatment settings that service these vulnerable and disadvantaged groups with very high smoking rates. Although some stop-smoking medicines are available at a subsidized cost through general practitioners, there are no easily accessible, face-to-face, cessation-specific services such as the National Health Service Stop Smoking Clinics which successfully reach lower SES smokers as in the UK [14]. The Australian Government recently committed to continuing substantial annual tobacco tax increases over 2016–2019 so that the real price of a pack of cigarettes increases to at least $40/pack. For those smokers that continue to smoke in the face of rising cigarette prices, experiences of financial stress are likely to be exacerbated [9] and we know that financially stressed smokers are less likely to quit [4]. As such, more targeted cessation support approaches are needed.

One avenue for reaching high numbers of smokers experiencing financial stress is social support services. These services are well placed to access traditionally “hard-to-reach” population groups of smokers, and addressing smoking as a social justice issue fits within their holistic care paradigm of social, financial and material support [15]. Preliminary work in this sector suggests that both clients and staff of SCSOs view addressing smoking in this setting as acceptable and feasible [16,17,18]. More research is needed to identify approaches that are effective for cessation in these settings, including assessing whether financial stress is significantly reduced as a result of cessation among this highly disadvantaged group. It may be that governments create programs to fund cessation support (e.g., provision of nicotine replacement therapy products) as an important component of delivering welfare services, or having existing health services (e.g., Quitline) more closely linked to this setting to ensure evidence-based cessation support is readily accessible when clients are willing to make a quit attempt.

This was a cross-sectional study and as such we cannot comment on any causal relationship between smoking and financial stress. Additionally, participants were recruited via non-probability-based convenience sampling while attending the community service organisation sites for the provision of emergency welfare aid and therefore may have been experiencing acutely high levels of financial stress compared to clients accessing other services within the community service sector. However, emergency relief is ranked by Australian community service organisations as one of the top three services in high demand by their clients [15]. Additionally, similar to the demographic profile of the current study, the report indicated that individuals with low income, who are unemployed or who have government income as their primary income source and who faced housing insecurity were overrepresented across all types of sector services [15].

One area that has not received much attention in the financial stress and smoking literature is the consideration of substance abuse and mental health comorbidities. Smoking is highly prevalent among those with severe mental health conditions (32%–90%) [19,20,21,22,23], as well as those attending substance abuse treatment (77%–95%) [24,25]. Indeed, smokers are more likely to spend money on alcohol and gambling [26], and this may contribute to financial stress. Individuals with very low SES often experience multiple layers of disadvantage and vulnerability. Within the Australian community service sector, mental health services continually report being unable to meet demand, and this service type is identified as a top priority area of need [15]. It is very likely that the current study sample included individuals with substance abuse and mental health comorbidities, however as this study employed a secondary analysis of two existing data sets that did not assess these variables, we were unable to include this in our analysis. Given the overlap of socioeconomic disadvantage, substance abuse and mental health comorbidities and the high prevalence of smoking across these markers of vulnerability, future research should consider controlling for substance abuse and mental health comorbidities in order to further advance our understanding of the role of financial stress within disadvantaged populations.

## 5. Conclusions

In conclusion, even among a highly socioeconomically disadvantaged sample with high levels of financial stress, smoking status is associated with increased experiences of deprivation. In particular, smokers are twice as likely as ex- and non-smokers experiencing hardship to go without meals or to pawn or sell their belongings due to a shortage of money. Given that individuals experiencing social and financial disadvantage are likely to engage with social and community service organisations, smoking cessation should be incorporated as part of the core business in this sector.

## Figures and Tables

**Table 1:** Demographic and smoking characteristics of the study sample (*N* = 1463).

		Smoking Status				
Characteristic	Class/Statistic	Non-Smoker (*n* = 305)	Ex-Smoker (*n* = 179)	Smoker (*n* = 979)	Total (*N* = 1463)	*p*-Value
Age	mean (SD)	41 (13)	43 (13)	38 (11)	39 (12)	<0.001
Gender	Male	85 (28%)	68 (38%)	451 (46%)	604 (41%)	<0.001
Education	Primary school	32 (10%)	13 (7.3%)	131 (13%)	176 (12%)	0.0006
	Secondary or less	173 (57%)	103 (58%)	616 (63%)	892 (61%)	
	Tertiary qualifications	100 (33%)	63 (35%)	232 (24%)	395 (27%)	
Indigenous status	Aboriginal and/or Torres Strait Islander	41 (13%)	24 (13%)	162 (17%)	227 (16%)	0.3009
Housing	Own house	43 (14%)	19 (11%)	38 (3.9%)	100 (6.8%)	<0.001
	Rental house	126 (41%)	65 (36%)	273 (28%)	464 (32%)	
	Supported accommodation/government housing	113 (37%)	83 (46%)	508 (52%)	704 (48%)	
	Homeless/Other	23 (7.5%)	12 (6.7%)	160 (16%)	195 (13%)	
Marital Status	Separated, divorced, single, widowed	207 (68%)	117 (65%)	807 (82%)	1131 (77%)	<0.001
	Married, de facto or living with partner	98 (32%)	62 (35%)	172 (18%)	332 (23%)	
Income Amount (AUD)	Less than $200 per week	56 (18%)	32 (18%)	246 (25%)	334 (23%)	0.0036
	Between $201 and $400 per week	156 (51%)	89 (50%)	504 (51%)	749 (51%)	
	More than $400 per week	76 (25%)	50 (28%)	172 (18%)	298 (20%)	
	Prefer not to answer	17 (5.6%)	8 (4.5%)	57 (5.8%)	82 (5.6%)	
Income Source	Paid employment (either full or part time)	28 (9.2%)	18 (10%)	35 (3.6%)	81 (5.5%)	<0.001
	Government pension or benefit/Centrelink	262 (86%)	152 (85%)	924 (94%)	1338 (91%)	
	Other	15 (4.9%)	9 (5.0%)	20 (2.0%)	44 (3.0%)	
No. of adults in house	median (IQR)	2 (1, 2)	2 (1, 2)	1 (1, 2)	1 (1, 2)	0.0055
No. of children in house	median (IQR)	1 (0, 2)	0 (0, 2)	0 (0, 2)	0 (0, 2)	0.1639

AUD: Australian Dollar; IQR: interquartile range; SD: standard deviation.

**Table 2:** Financial stress scores (mean, 95% Confidence Interval, CI) for non-, ex-, and current smokers.

Smoking Group	Crude	Adjusted ^1^
Non-smoker	4.8 (4.6, 5)	4.9 (4.6, 5.1)
Ex-smoker	5 (4.7, 5.2)	5.1 (4.8, 5.4)
Current smoker	5.5 (5.4, 5.6)	5.5 (5.4, 5.7)

^1^ Adjusted for age, gender, education, Indigenous status, income, number of adults in house, number of children in house.

**Table 3:** Adjusted ^1^ comparison of financial stress individual indicators for non-, ex-, and current smokers.

	Smoker vs. Non		Smoker vs. Ex		Ex vs. Non		
Financial Stress	OR (95% CI)	*p*	OR (95% CI)	*p*	OR (95% CI)	*p*	Overall *p*
Could not pay bills	0.89 (0.6, 1.2)	0.4628	0.70 (0.5, 1.0)	0.0850	1.27 (0.8, 2.0)	0.3131	0.2099
Could not pay mortgage/rent	1.32 (1.0, 1.8)	0.0571	1.35 (1.0, 1.9)	0.0939	0.98 (0.7, 1.5)	0.9049	0.0701
Pawned/sold something	2.79 (2.1, 3.7)	<0.0001	1.92 (1.4, 2.7)	0.0002	1.46 (1.0, 2.2)	0.0586	<0.0001
Went without meals	2.19 (1.7, 2.9)	<0.0001	1.99 (1.4, 2.8)	<0.0001	1.10 (0.7, 1.6)	0.6307	<0.0001
Could not heat/cool home	1.12 (0.8, 1.5)	0.4306	1.22 (0.9, 1.7)	0.2589	0.92 (0.6, 1.4)	0.6637	0.4548
Asked for help from family/friends	1.77 (1.3, 2.4)	0.0003	1.35 (0.9, 2.0)	0.1235	1.31 (0.9, 2.0)	0.2086	0.0012
Asked for help from welfare/community association	2.29 (1.5, 3.5)	<0.0001	1.70 (1.0, 2.9)	0.0498	1.35 (0.8, 2.4)	0.3007	0.0004
Raise emergency cash	0.77 (0.4, 1.5)	0.4397	0.68 (0.3, 1.6)	0.3866	1.14 (0.4, 3.1)	0.8048	0.5620

^1^ Adjusted for age, gender, education, indigenous status, income, number of adults in house, number of children in house. OR, odds ratio.
